# The Rise and Fall of Hyaluronan in Respiratory Diseases

**DOI:** 10.1155/2015/712507

**Published:** 2015-09-10

**Authors:** Mark E. Lauer, Raed A. Dweik, Stavros Garantziotis, Mark A. Aronica

**Affiliations:** ^1^Pediatric Institute, Cleveland Clinic, Cleveland, OH 44195, USA; ^2^Department of Biomedical Engineering, Cleveland Clinic, Cleveland, OH 44195, USA; ^3^Department of Pathobiology, Cleveland Clinic, Cleveland, OH 44195, USA; ^4^Department of Pulmonary and Critical Care Medicine, Cleveland Clinic, Cleveland, OH 44195, USA; ^5^National Institute of Environmental Health Sciences, Research Triangle Park, NC 27709, USA; ^6^Respiratory Institute, Cleveland Clinic, Cleveland, OH 44195, USA

## Abstract

In normal airways, hyaluronan (HA) matrices are primarily located within the airway submucosa, pulmonary vasculature walls, and, to a lesser extent, the alveoli. Following pulmonary injury, elevated levels of HA matrices accumulate in these regions, and in respiratory secretions, correlating with the extent of injury. Animal models have provided important insight into the role of HA in the onset of pulmonary injury and repair, generally indicating that the induction of HA synthesis is an early event typically preceding fibrosis. The HA that accumulates in inflamed airways is of a high molecular weight (>1600 kDa) but can be broken down into smaller fragments (<150 kDa) by inflammatory and disease-related mechanisms that have profound effects on HA pathobiology. During inflammation in the airways, HA is often covalently modified with heavy chains from inter-alpha-inhibitor via the enzyme tumor-necrosis-factor-stimulated-gene-6 (TSG-6) and this modification promotes the interaction of leukocytes with HA matrices at sites of inflammation. The clearance of HA and its return to normal levels is essential for the proper resolution of inflammation. These data portray HA matrices as an important component of normal airway physiology and illustrate its integral roles during tissue injury and repair among a variety of respiratory diseases.

## 1. Introduction

**Figure 1:**
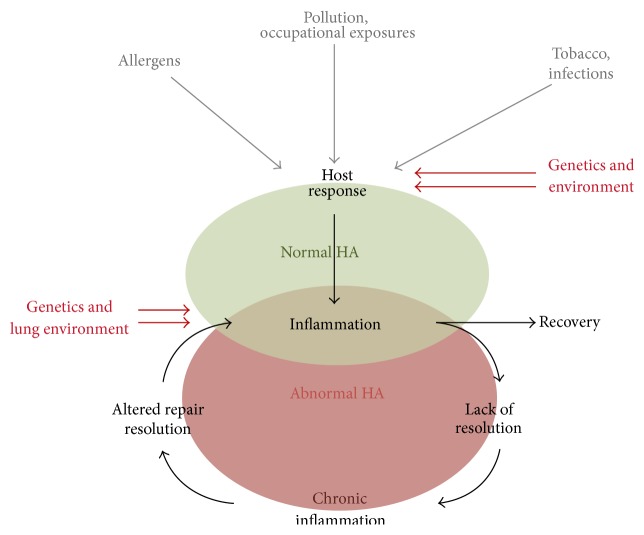
Overview of hyaluronan in respiratory disease: the lung is continuously exposed to external stimuli which can then impact HA synthesis and turnover. Factors such as type of stimuli, genetics, and the lung environment itself determine if resolution or persistent inflammation and HA changes persist.

Considerable progress has been made over the past few decades in our understanding of the role of hyaluronan (HA) in pulmonary health and disease. Once thought to be an inert molecule of the extracellular matrix, a picture has emerged of HA as an important regulator of inflammation, airway hyperresponsiveness (AHR), edema, and fibrosis in the lung. This image has been made clearer by a significant number of investigations into a wide variety of different pulmonary diseases, environmental effects, and animal models of lung injury, which are summarized in this review (Figure 1).

HA is a major component of extracellular matrices (ECM) in every major organ system [1, 2]. It is a very large (>2500 kDa), unsulfated glycosaminoglycan, unattached to a core protein, though associated with several HA binding proteins and receptors that expand its repertoire of functions [3, 4]. HA is extruded from the cell surface by three membrane-bound HA synthases (HAS1, HAS2, and HAS3) that utilize cytosolic UDP-N-acetyl-D-glucosamine (UDP-GlcNAc) and UDP-D-glucuronate (UDP-GlcUA) as substrates to form the repeating disaccharide unit *β*1,3*-N-*GlcNAc linked *β*1,4 to GlcUA [5]. The turnover of HA varies from tissue to tissue and is mediated by a family of hydrolytic, lysosomal enzymes known as hyaluronidases [6]. As murine knockout models of airway injury have shown, the clearance of HA, and its return to normal levels, is critical for the resolution of inflammation [7]. Under normal conditions, HA is synthesized as a high molecular weight (HMW) polysaccharide but can be degraded into smaller bioactive fragments during inflammatory and pathological processes [8, 9]. The only covalent modification known to occur on HA is a transesterification reaction with a C-terminal aspartate residue (Asp^702^) of an inter-alpha-inhibitor heavy chain to the 6th hydroxyl of GlcNAc on HA via the enzyme tumor-necrosis-factor-stimulated-gene-6 (TSG-6) [10–14]. This reaction occurs during pathological and developmental processes and has been found in inflamed and remodeling lungs [15–17].

The present review provides a survey of HA-related data in the area of pulmonary pathobiology with an emphasis upon its expression, distribution, and turnover in a variety of respiratory disorders and conditions from both human subjects and animal models. The review is divided into three major headings: (a) environmental and occupational exposure, (b) human respiratory diseases, and (c) animal models of pulmonary injury. For a review of HA receptors and binding proteins in lung pathobiology the reader is advised to consult the review by Lennon and Singleton [18]. This review, and another [19], includes an overview of the therapeutic applications of HA in the lung. Whenever possible, actual concentrations of HA and *p* values were listed from the original sources. Units of measurement, such as *μ*g/L of HA in bronchoalveolar lavage fluid (BAL), were standardized throughout the text. The type of assay used to quantify HA was noted in most instances, and specific information on the assay was stated when possible (i.e., sandwich versus competitive ELISA-like assays). The most common methods presented in this review are radiometric or ELISA-like assays involving an HA binding protein derived from cartilage. For an overview and comparison of the sensitivity, specificity, and molecular weight accuracy of commercially available ELISA-like assays for HA analysis please see our review [20]. It should be noted that methods utilizing an HA binding protein do not distinguish between HA modified with heavy chains and HA without this modification. At times, data had to be estimated from graphs. In such instances, the data was presented as an approximation (i.e., ~10 *μ*g/L).

## 2. Environmental and Occupational Exposure

### 2.1. Farmer's Lung

Farmer's Lung is a type of alveolitis caused by a type III hypersensitivity reaction induced by the inhalation of mold derived from plant material in the agricultural industry [21]. Inflammation occurs in the alveolar wall in response to an IgG-allergen mediated immune response, causing edema and loss of pulmonary function in severe cases. Bjermer et al. examined ten patients during acute episodes of farmer's lung [21]. Impaired diffusion capacity (on average 51% of predicted) was associated with elevated levels of HA (mean concentration 547 *μ*g/L) in bronchoalveolar lavage (BAL) fluid compared to healthy controls (15 *μ*g/L) (as determined by a radiometric assay using an HA binding protein labeled with iodine-125). HA levels declined (154 *μ*g/L) during the 4–10-week recovery phase, nearly to normal levels at clinical remission 14 months after admission, though slightly elevated concentrations of HA were observed in about half of the subjects. Similar findings were observed in a separate study [22], and HA in BAL fluid (radiometric assay) was found to distinguish farmers with allergic alveolitis from farmers with asymptomatic alveolitis [23]. These data demonstrated that the accumulation of HA in farmer's lung was associated with the progression of the disease, suggesting the possibility that HA in the smaller airways may contribute to edema and impaired gas exchange by its relatively high hydration capacity.

### 2.2. Swine Confinement Workers

Larsson et al. tested the hypothesis that swine confinement workers have signs of airway inflammation, alterations of lung function, and bronchial responsiveness [24]. These workers are exposed to dust containing feed, fecal, and dander particles and develop airway symptoms, including cough, phlegm, wheeze, tightness of chest, and slight decreases in FEV_1_, which had been reported [25, 26]. In Larsson's study, lung function, bronchial reactivity, and several tests for inflammation were performed on 20 randomly selected nonsmoking swine confinement workers who regarded themselves as healthy. While lung function and bronchial reactivity were not different from the urban reference group, inflammatory markers, such as elevated BAL leukocyte counts, elevated antibodies against swine dander, dust and feed, and elevated BAL levels of HA (37 *μ*g/L compared to 27 *μ*g/L in the reference group) were observed (*p* < 0.01) (radiometric assay). The authors concluded that signs of airway inflammation could be altered in pig farmers without alteration in lung function or bronchial reactivity. HA in BAL was found to be within normal limits in a similar study of asymptomatic dairy farmers [27] (radiometric assay).

### 2.3. Swedish Wood Trimmers

Workers in the logging industry are routinely exposed to mold released into the air during the harvest and transport of trees. Some of these workers develop allergic alveolitis as a result of this exposure [28]. In a study by Johard et al., signs of alveolitis were investigated in a population of Swedish wood trimmers [29]. Nineteen nonsmoking workers were subdivided into two groups, with or without serological antibodies against mold. While no difference was found in lung function (spirometry and diffusion capacity) among these subjects, BAL levels of HA were significantly elevated (42 *μ*g/L compared to 27 *μ*g/L in the controls) (*p* < 0.001) (radiometric assay). HA levels were not different between seropositive and seronegative workers, indicating that the elevated antibodies against mold did not predict increased risk for the development of airway inflammation. In a related rat study, this group also reported that the intratracheal instillation of sawdust, itself, resulted in increased inflammation and elevated HA levels in BAL [30] (radiometric assay).

### 2.4. Firefighters

Firefighters are exposed to toxic fire gases and other combustion products from their occupation which may cause acute and chronic respiratory symptoms [31, 32]. Bergström et al. tested the hypothesis that firefighters might develop inflammatory changes in their lower airways as a result of this exposure [33]. BAL was obtained from 13 nonsmoking firefighters and the results were compared to a reference group of 112 nonsmoking healthy control subjects. Elevated HA levels were observed in firefighters (27.7 *μ*g/L) compared to the control population (10 *μ*g/L) (*p* < 0.05) (radiometric assay). While no attempt was made to correlate the extent and timing of smoke exposure with HA levels, their data suggests that long-term occupational exposure results in inflammation that corresponds with elevated HA levels.

### 2.5. Asbestos

Asbestos is derived from silicate minerals and has been used to provide electrical and building insulation due, in part, to its resistance to fire [34]. Asbestos is composed of fibrous crystals that can accumulate in the air and cause lung injury as a result of inhalation, including lung cancer, mesothelioma, pleural plaques, pleural effusion, and asbestosis [35]. In a study conducted by Cantin et al., HA concentration was measured in the BAL of 27 workers from asbestos mills and mines, 9 without asbestosis, and 18 with asbestosis [36]. Mean HA levels were found to be 53.9 *μ*g/L in control subjects, 67.5 *μ*g/L in asbestos-exposed workers without asbestosis, and 206 *μ*g/L in workers with asbestosis (*p* < 0.05) (radiometric assay). This study also examined HA in the BAL of asbestos-exposed sheep. Asbestos was applied by intratracheal infusions of chrysotile at 10 mg or 100 mg doses every 10 days for 39 months. Mean HA levels were found to be 34.7 *μ*g/L in control (PBS) sheep, 83.0 *μ*g/L in sheep treated with the 10 mg dose, and 248.0 *μ*g/L in the sheep treated with the 100 mg dose (*p* < 0.05). These data indicate that BAL HA levels correspond with the extent of lung damage by asbestos and with the amount of exposure to asbestos. A separate study also observed that serum HA levels corresponded to malignancies caused by asbestos exposure [37] (radiometric assay).

### 2.6. Flour Dust

Flour dust exposure can lead to the development of an IgE-mediated sensitization to flour proteins causing asthma and rhinitis in the baking industry in a condition known as baker's asthma [37]. Brisman et al. analyzed indices of nasal airway inflammation in bakers, seeking to relate these to nasal symptoms and exposure to airborne flour dust [38]. Twelve flour-exposed bakers participated in this study and were examined by nasal lavage, visual inspection, a test for mucociliary clearance, and nasal peak expiratory flow. A significant correlation between nasal lavage HA levels and nasal mucociliary clearance was observed (radiometric assay). Two atopic individuals had the highest levels of HA in the nasal lavage and there was a positive correlation between the cumulative dose of flour dust and HA nasal lavage levels. Furthermore, HA nasal lavage levels correlated with the number of years the subjects worked as bakers. These data indicate that a baker's occupational exposure of flour dust can cause nasal mucosal inflammation that is associated with elevated levels of HA in nasal secretions.

### 2.7. Conclusions

**Figure 2:**
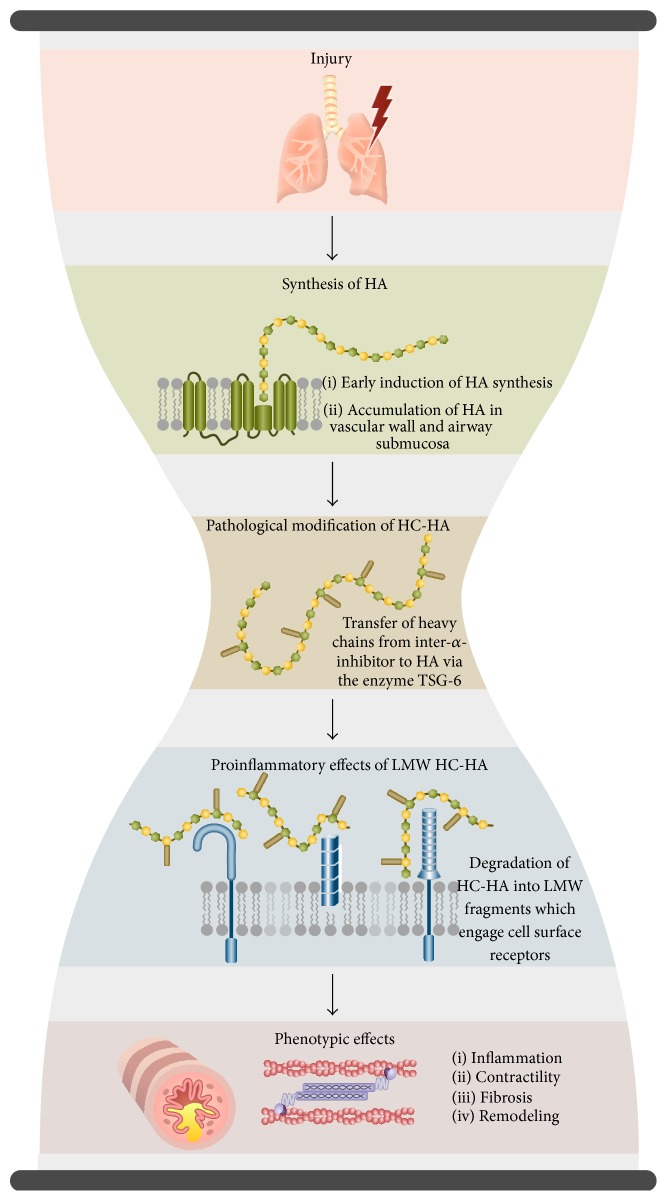
Mechanistic role of HA in the response to lung injury: lung injury leads to the synthesis of HA that accumulates in the peribronchial and perivascular spaces. The ongoing inflammation leads to the generation of heavy-chain-HA (HC-HA) complexes mediated via TSG-6 which is a bottleneck in the pathological transformation of HA matrices. These HC-HA complexes can be degraded into smaller LMW fragments which engage cell receptors such as CD44, TLR4, and TLR2 and create downstream biological effects like fibrosis, AHR, and inflammation.

These studies indicate that (i) elevated levels of HA in BAL fluid are associated with a variety of environmental and occupational airway injuries. (ii) HA levels correspond to the extent of exposure and lung injury. And (iii) elevated HA levels in BAL may be present even in the absence of obvious lung disease. As shown in Figure 1, one of the host responses of the airways to occupational and environmental exposures is the production of HA in the lung tissue and pulmonary secretions. If the exposure continues, and inflammation fails to resolve, abnormal matrix remodeling occurs, including the chronic synthesis of HA, its modification with the heavy chains of inter-alpha-inhibitor, and the production of proinflammatory HA fragments which exacerbates the inflammatory and fibrotic stimuli [9, 39–45] (Figure 2). It should be noted that the role of heavy chain modified HA fragments in directing inflammatory events has not yet been elucidated.

## 3. Human Respiratory Diseases

### 3.1. Smoking

Tobacco smoking is a major risk factor for the development of several lung diseases most notably lung cancer as well as chronic obstructive pulmonary disease (COPD) which includes emphysema and chronic bronchitis. One of the early findings, by McDevitt et al. [46], was that the gas phase of cigarette smoke, introduced into solutions of HA by the method of Kew et al. [47], led to a marked reduction in HA viscosity and size in a time-dependent manner. Dimethyl sulfoxide, a scavenger of hydroxyl radicals, inhibited this degradation, suggesting that oxidative damage by free radicals in the gas phase of cigarette smoke was the mechanism by which HA was degraded. Bracke et al. confirmed these observations in a mouse model of cigarette smoke-exposed mice [42] (see animal model section below for more information). In a study conducted by Sköld et al. [48], smoking cessation was found to result in a transient induction of HA accumulation in the BAL 1 month (38.8 *μ*g/L), 3 months (34.0 *μ*g/L), and 6 months (37.5 *μ*g/L) following smoking cessation, compared to smokers before cessation (28.6 *μ*g/L) and nonsmokers (10.0 *μ*g/L) (*p* < 0.01 to 0.001) (radiometric assay). This data implies that the induction of HA following smoking cessation may have a reparative effect on lung pathology caused by smoke injury, while smoking itself can degrade HA into LMW forms that may promote inflammation [9].

### 3.2. COPD

Chronic obstructive pulmonary disease (COPD) is a progressive lung disease characterized by fixed airway obstruction that results in shortness of breath, cough, and sputum production and is typically caused by tobacco smoking [49]. In one study, COPD patients had elevated (~27 *μ*g/L) levels of HA in the BAL compared to normal nonsmokers (~17 *μ*g/L) (*p* < 0.01) [50] (radiometric assay). Additionally, COPD patients with lower pulmonary function measurements had higher levels of HA in BAL than COPD patients with relatively normal pulmonary function. Similarly, in a separate study, HA levels were higher in the sputum of COPD patients (234 *μ*g/L) than healthy smokers (66 *μ*g/L) (*p* < 0.005) [51] (ELISA-like assay). Furthermore, COPD patients with higher levels of sputum HA had lower FEV_1_, and higher inflammatory markers, than COPD patients with moderate levels of HA. These data indicate that there is a relationship between HA levels in COPD sputum and BAL that corresponds with disease severity.

### 3.3. Asthma

Asthma is a chronic inflammatory disease characterized by bronchial wall basement membrane thickening, airway smooth muscle hypertrophy, mucous gland hypertrophy, vascular dilation, and airway epithelial damage [52]. The original report of HA in human asthma found HA to be the only glycosaminoglycan present in the pulmonary secretions (BAL) from 4 patients with severe asthma [53]. Later, it was determined that HA levels in BAL were significantly increased in patients with persistent asthma who were atopic (32 *μ*g/L) and nonatopic (21 *μ*g/L) in comparison to control subjects (0 *μ*g/L) and patients with mild intermittent asthma (0.5 *μ*g/L) (*p* < 0.001) [54] (radiometric assay). Liang et al. isolated and cultured fibroblasts from endobronchial biopsies of 21 asthmatics, and 25 healthy controls, and examined the cells for HA production [43]. Baseline (unstimulated) HA production by airway fibroblasts was significantly (*p* < 0.05) higher in the asthmatic population (~900 *μ*g/L) compared to healthy controls (~200 *μ*g/L) (ELISA-like assay). Furthermore, the average HA size was lower in MW for the asthmatic (~800 kDa) population than controls (~1500 kDa). This was accompanied by a marked increase in HAS2 gene expression in asthmatic (~18-fold) compared to control (~3-fold) fibroblasts (*p* < 0.05). In another study by Ayars et al., patients with severe, steroid-dependent asthma received either mepolizumab (anti-IL-5 antibody) or placebo in a randomized, double-blind, placebo-controlled study [55]. Sputum HA was measured after 16 weeks of treatment. A significant decrease in sputum HA was observed in the mepolizumab treatment group (97 *μ*g/L) compared to the placebo group (266 *μ*g/L) (*p* = 0.007), which correlated with improvements in percent forced expiratory volume in 1 s (FEV_1_%) (*p* = 0.001) (competitive ELISA-like assay). In summary, HA is an important component of airway secretions, and cultured fibroblasts, from asthmatics that corresponds to disease severity and pulmonary function.

### 3.4. Sarcoidosis

The pulmonary manifestation of sarcoidosis is the accumulation of granulomas in the interstitium, including the alveoli, small airways, and blood vessels [56]. The disease progresses to fibrosis in a small percentage (5–15%) of cases. Hallgren et al. found HA (16 *μ*g/L) in the BAL of 23 patients with sarcoidosis, though it was undetectable in smoking or nonsmoking healthy volunteers [57] (radiometric assay). Serum HA levels were normal, but patients with abnormal lung function (spirometry) had HA BAL concentrations six times higher than patients with normal vital capacity. In a separate study, Eklund et al. found HA BAL levels from 23 sarcoidosis patients at 12 *μ*g/L on average [58] (radiometric assay). HA levels positively correlated with the numbers of BAL leukocytes. Bjermer et al. identified a strong correlation between BAL HA and mast cell levels which correlated inversely with lung volume [59] (radiometric assay). Macrophages and granulocyte counts were not related to BAL HA levels or indicators of lung disease, though lymphocyte counts were significantly (*p* < 0.001) elevated and corresponded to mast cells and HA levels. Blaschke et al. demonstrated that elevated levels of other extracellular matrix components, including fibronectin and type III procollagen peptide, correlated with elevated levels of HA (39 *μ*g/L compared to control values of 25 *μ*g/L) in the BAL of patients with sarcoidosis (*p* < 0.001) [60] (radiometric assay). In summary, HA BAL levels are elevated in patients with sarcoidosis and correspond to decreased lung function, increased leukocyte counts, and increased extracellular matrix components.

### 3.5. Idiopathic Pulmonary Fibrosis

Idiopathic pulmonary fibrosis is a disease of the lung interstitium that involves fibrosis within alveolar tissue, small airways, and blood vessels [61]. Bjermer et al. found elevated levels of HA (46 *μ*g/L) in the BAL fluid of 22 patients with idiopathic pulmonary fibrosis compared to 21 control subjects (9 *μ*g/L) (*p* < 0.001) [62] (radiometric assay). Serum HA levels were normal, but elevated neutrophil and lymphocyte counts correlated with the increased levels of HA. Patients with deterioration of lung function and radiographic progression had higher BAL levels of HA than in patients whose disease was stable (*p* < 0.01). This study was largely substantiated by a separate study conducted by Milman et al. who expanded it to include correlations with procollagen type III aminoterminal peptide [63]. In a histological examination of HA by Garantziotis et al., HA was found to colocalize with inter-alpha-inhibitor in fibroblastic foci of patients with usual interstitial pneumonitis, implying that the HA present in these regions is likely to be covalently modified by heavy chains from inter-alpha-inhibitor [17].

### 3.6. Idiopathic Pulmonary Hypertension

Idiopathic pulmonary arterial hypertension (IPAH) is a progressive disease that leads to deterioration in cardiopulmonary function and premature death from right ventricular failure [64]. The pathogenesis of IPAH includes cell proliferation, vascular remodeling, and inflammatory cell recruitment. Papakonstantinou et al. investigated total glycosaminoglycan content between IPAH and control donor lungs and found that only HA had elevated levels associated with IPAH [65]. The relative HA levels in IPAH lung tissue (78.6 *μ*g) were greater than the amount of HA in control donor lung tissue (43.2 *μ*g) (*p* < 0.01), consistent with the observation that HAS1 gene and protein expression was elevated in the IPAH cohort while Hyal1 gene expression was significantly decreased (*p* < 0.05) (ELISA-like assay). HAS1 protein localized to pulmonary arterial smooth muscle cells in IPAH lung tissue and increased HA deposition was observed in remodeled pulmonary arteries. In a separate study, Aytekin et al. demonstrated that plasma HA levels were markedly elevated in IPAH (325 *μ*g/L) compared with controls (28 *μ*g/L) (*p* = 0.02) [66] (competitive ELISA-like assay). Cultured, and unstimulated, pulmonary arterial smooth muscle cells from IPAH patients secreted higher levels of HA into conditioned media (12 *μ*g/mL) compared to control cells (6 *μ*g/mL) (*p* = 0.04). This HA was in the form of “cable” structures that promoted the adhesion of mononuclear cells, comparing their adhesion to pulmonary arterial smooth muscle cells from IPAH (9.5 × 10^4^ cells bound) and control subjects (3.0 × 10^4^ cells bound) (*p* = 0.01). This same group also observed that the HA in IPAH lungs is a pathological form of HA covalently modified with heavy chains from inter-alpha-inhibitor [16]. Heavy chain modified HA (HC-HA) was found within regions of vascular modeling, including plexogenic lesions. Inflammatory cells colocalized within these matrices in regions of vascular pathology in IPAH lung tissue, raising the possibility that HC-HA may direct inflammatory events that cause vascular remodeling in IPAH.

### 3.7. Lung Transplant

Rao et al. investigated HA BAL and plasma levels from 57 lung transplant recipients as a marker of early posttransplantation infection and acute cellular rejection [67]. Mean BAL HA levels in recipients with clinically stable conditions was 33.5 *μ*g/L (radiometric assay). Mild rejection did not result in significant BAL HA levels, though it was slightly higher with infection (51.10 *μ*g/L) (*p* = 0.036). Moderate to severe rejection resulted in significantly elevated BAL HA levels (295.0 *μ*g/L) (*p* = 0.0001) and the highest levels were found in patients with diffuse alveolar damage (392 *μ*g/L). Mean plasma HA levels in clinically stable recipients were 59.60 *μ*g/L and were elevated in severe rejection (112.0 *μ*g/L) and diffuse alveolar damage (169.20 *μ*g/L) and even higher in recipients with acute respiratory infection (191.0 *μ*g/L). These observations were substantiated and expanded by Riise et al. [68, 69].

### 3.8. Bronchiolitis Obliterans

One of the major causes of lung transplant rejection is the onset of bronchiolitis obliterans syndrome (BOS) which is characterized by irreversible limitations of airflow associated with small airway fibrosis [70]. Todd et al. found elevated levels of HA within the intraluminal fibrous tissue of patients with BOS [71]. This corresponded with elevated expression (2-3-fold) of HAS1-3 in whole lung tissue from BOS compared to control subjects (*p* < 0.05). HA BAL levels were elevated in BOS subjects (107.91 *μ*g/L) compared to controls (28.97 *μ*g/L) (*p* < 0.0001) and remained steady between different grades of BOS. Furthermore, HA plasma levels were elevated in early or severe onset BOS subjects (90.37 *μ*g/L) compared to patients who had remained BOS free for at least 5 years (44.42 *μ*g/L) (*p* = 0.03) (sandwich ELISA-like assay).

### 3.9. Conclusions

(i) In normal tissues, HA matrices are primarily located within (a) the airway submucosa, (b) the walls of pulmonary vasculature, (c) and to a lesser extent, alveoli. (ii) During pulmonary injury and repair, there is increased synthesis of HA matrices in these regions that colocalizes with inflammatory cells and likely influences their activation. (iii) The HA that accumulates in these regions is often covalently modified with heavy chains from inter-a-inhibitor which significantly promotes leukocyte adhesion to HA matrices [15–17, 72, 73] (Figure 2). (iv) Elevated HA BAL levels correspond with the extent of lung injury while HA serum levels do not always correlate with lung injury.

## 4. Animal Models of Pulmonary Injury

### 4.1. Asthma

HA deposition and correlation with inflammation have been described in the ovalbumin [74], cockroach antigen [75], and* Aspergillus fumigatus* [76] murine models of allergic inflammation. In the ovalbumin model described by Cheng et al., the accumulation of HA within BAL was a relatively early event, occurring within the first 24 hrs after allergen challenge (~25 *μ*g/L compared to ~10 *μ*g/L in naïve controls) (sandwich ELISA-like assay). HA BAL levels peaked at day 8 (~125 *μ*g/L), corresponding with elevated inflammatory cell counts in the BAL. Induction of whole lung HAS1 and HAS2 gene induction peaked (~20-fold above naïve values) within the first 2–4 hrs and steadily declined to almost normal levels by the end of 24 hrs. The accumulation of HA in the lung tissue was evident 12 hrs after allergen challenge (~140 *μ*g/g dry weight compared to ~75 *μ*g/g in naïve controls), peaked at 6 days (~375 *μ*g/g), and steadily declined to lower levels 6 weeks later (~150 *μ*g/g) (Fluorophore-Assisted-Carbohydrate-Electrophoresis (FACE) analysis). Elevated peribronchial distribution of HA was apparent 12 hrs after allergen challenge and colocalized with eosinophil distribution 2 days later (HABP fluorescence microscopy). Similar results were confirmed and expanded in cockroach [75] and fungal models of allergic airway inflammation [76]. The HA that accumulated in murine lungs following ovalbumin challenge was covalently modified with heavy chains from inter-alpha-inhibitor [15]. TSG-6 −/− mice, which lack the ability to transfer heavy chains to HA, developed less inflammation, lower AHR, and lower levels of HA in response to allergen challenge, implying that HC-HA is an important factor in allergic inflammation [15]. Lower levels of lung HA in TSG-6 −/− mice (FACE and HAPB fluorescence microscopy) subjected to allergen induced asthma may be caused by the ability of TSG-6 to not only transfer heavy chains to HA but also regulate HA accumulation [77].

### 4.2. Bleomycin

The bleomycin model of pulmonary fibrosis is based upon a side-effect of its use as a chemotherapeutic agent for the treatment of several cancers [78]. Bleomycin exerts its antibiotic and tumorigenic effect by inducing DNA strand breaks, though the mechanism whereby it induces lung injury is not fully understood [79]. In rodents, bleomycin induces acute alveolitis with interstitial edema and fibrosis. Using a biotinylated HA binding protein to probe rat lung tissue sections, Nettelbladt et al. observed that HA accumulated in the edematous alveolar septa 4 days after the intratracheal administration of bleomycin [80]. Lymphocytes were present in the interstitial cellular infiltrate. In control rat lungs, HA was not seen in the alveolar tissue but was confined to peribronchial and perivascular spaces. Ten and twenty days after administration of bleomycin, macrophage infiltration was observed, as well as proliferating fibroblasts and collagen deposition in the alveolar tissue. At these later time points, HA deposition became less prominent in the alveolar interstitial tissue but more distinctly located around proliferating fibroblasts. The authors noted increased lung water content that peaked 4 days after bleomycin treatment and speculated that increased levels of HA might contribute to the water accumulation. This same group also noted that the accumulation of HA in the BAL of rats treated with bleomycin was relatively small (0.2-0.3 × 10^6^ Daltons) and did not respond to high-dose corticosteroid treatment [81]. In a separate study in hamsters, Bray et al. observed that total lung HA levels peaked 6 days after administration of bleomycin and was 14.6-fold higher than untreated levels (autoradiography) [82]. These levels sharply dropped on day 7 and steadily declined to approximately double control values by day 17. Total levels of lysosomal hyaluronidases were increased (673 units compared to 506 units in control lungs) in the injured lungs, even at the peak of HA accumulation on day 6, indicating active turnover of HA. It was also observed that maximal HA content occurred prior to the rise in collagen and elastin biosynthesis, suggesting that HA acts as an acute phase molecule that may direct subsequent fibrotic events. Further evidence for an early role of HA during bleomycin-induced alveolitis was obtained by Nettelbladt et al. who demonstrated that HA induction was apparent within 24 hrs of bleomycin treatment, much earlier than the fibrotic stage that occurs several days later (radiometric assay) [83]. Garantziotis et al. observed that bikunin −/− mice, deficient in their ability to covalently modify HA with heavy chains from inter-alpha-inhibitor, demonstrated deficient lung angiogenesis after bleomycin exposure [17]. Teder et al. observed that CD44 −/− mice succumbed to unremitting inflammation following bleomycin lung injury, characterized by the accumulation of HA fragments at the site of tissue injury and impaired clearance of macrophages, neutrophils, and lymphocytes [84]. Dygai et al. observed that the intranasal application of hyaluronidase, immobilized on polyethyleneoxide, did not modify the inflammatory process or deposition of collagen fibrils in the lung parenchyma [85]. Studies by others have substantiated, and expanded, these observations [86–92].

### 4.3. Elastase

The intratracheal administration of elastase remains a common and convenient method for the induction of emphysema-like airway pathology, including the augmentation of airspaces, inflammatory cell influx into the lungs, and systemic inflammation [93]. In two early studies, Moczar et al. demonstrated that cultured lung explants from hamsters, intratracheally injected with elastase, demonstrated significantly enhanced incorporation of ^14^C-glucosamine into HA [94]. In a separate study by Cantor et al., coadministration of hyaluronidase with elastase resulted in significantly greater airspace enlargement than hamsters treated with elastase alone [95]. Rescue by the intratracheal administration of HA immediately after elastase instilment resulted in a marked decrease in airspace enlargement. When HA was administered 1 or 2 hrs before elastase administration, rescue of airspace enlargement was retained [96]. When HA was administered 1 or 2 hrs after elastase administration, rescue was compromised. Scuri et al. demonstrated a protective effect on bronchoconstriction of inhaled HA against elastase-induced injury in sheep, where 200 kDa HA had significantly greater protective effect than 70 kDa HA [97]. This protective effect was also observed in the 150–300 kDa range which was found to be more dependent upon dosage rather than MW [98]. Studies by others have substantiated and expanded these observations [99–101].

### 4.4. Hyperoxia

Preterm birth by cesarean section often results in an imbalance of fluid secretion and absorption in the lungs that results in interstitial edema which is treated by oxygen supplementation and/or ventilator support which can exacerbate pulmonary fluid retention [102]. Juul et al. demonstrated that the HA content of untreated neonatal rat pups decreased over the first 10 days of life while pups housed under hyperoxic conditions exhibited a time-dependent increase in both lung HA and lung weight [103]. This HA accumulated in the perivascular regions of medium sized arteries and in the alveolar walls. A similar study by Johnsson et al. confirmed and expanded these observations in rabbit pups, delivered by cesarean section 1 or 2 days before term [104]. Though this report did not observe a time-dependent decrease in HA content of pups housed under room air conditions, continuous exposure to hyperoxia resulted in significantly elevated levels of lung HA concentration 6 days after term. This increase was accompanied by significantly elevated wet/dry lung weight ratios. Increased HA deposition was observed in alveoli, arterioles, and bronchiole of pups housed under hyperoxic conditions. The extent to which the elevated HA levels, induced by hyperoxia, contribute to edema is not yet known.

### 4.5. Cigarette

Tobacco smoke contains >7000 chemicals, including cyanide, benzene, formaldehyde, methanol, acetylene, and ammonia [105]. At least 70 of these chemicals are known carcinogens and many of them cause heart and lung diseases in addition to cancer [106]. Mice exposed to cigarette smoke for 4 weeks (subacute) or 24 weeks (chronic) demonstrated higher levels of staining for HA in alveolar walls for both time points [42]. This was in contrast to the deposition of collagen and fibronectin which were only elevated at the chronic time point. A modest (~25%) increase of HA levels in total lung tissue was observed in the smoke-exposed mice at 4- and 24-week time points. The size of this HA was significantly smaller (average MW about 70 kDa) than HA without smoke exposure (broad range of 250–1000 kDa), though it remains to be determined whether the MW redistribution was caused by oxidative damage from smoke exposure itself or from downstream inflammatory effects. Cigarette smoke induced HAS3 gene expression (~40%) and decreased HAS1 expression (~30%) while not significantly effecting HAS2 gene expression. Two separate studies demonstrated a therapeutic effect for inhaled aerosolized HA (150 and 1600 kDa) in a mouse and rat model of cigarette smoke exposure [107, 108]. These therapeutic effects included significantly less neutrophil infiltration, lung edema, airway apoptosis, and mucus plugging.

### 4.6. Ozone

Ozone exposure leads to oxidative stress-induced inflammation of the airways, epithelial injury, and AHR which peaks at 24 hours after exposure. In a study conducted by Garantziotis et al., ozone exposed mice demonstrated enhanced AHR associated with elevated levels (~40 *μ*g/L compared to undetectable levels in normal air exposed mice) of HA in BAL [39] (ELISA-like assay). CD44 −/− (a major receptor for HA) and bikunin −/− (unable to make HC-HA) mice showed even higher levels of elevated HA in response to ozone exposure (~100 *μ*g/L) but had significantly lower levels of AHR compared to WT mice. Mice pretreated with HA binding protein were protected from developing ozone-induced AHR. LMW HA exacerbated AHR in response to ozone treatment while HMW HA alleviated it. An allergic model of asthma was also found to exacerbate AHR and HA BAL levels in response to ozone treatment [109]. Other studies have substantiated and expanded these observations [40, 110–112].

### 4.7. Radiation

The pathological effect of radiation on the respiratory system is complex, involving the death of lung cells and the mounting of an inflammatory response [113]. The two major functional outcomes of radiation damage on the respiratory system include radiation pneumonitis and radiation fibrosis [113]. In a rat model of bilateral radiation-induced lung disease, Rosenbaum et al. found elevated levels of HA in serum (5.5-fold) and BAL (1.5-fold) 4 weeks after irradiation, during peak alveolitis [114]. Elevated levels of HA were not observed at earlier (2 weeks) or later (6–20 weeks) time points; thus serum HA levels appear to be a poor predictive indicator of radiation-induced pneumonitis. Histological staining demonstrated that HA accumulated in the intra-alveolar edema fluid but not the alveolar walls. Administration of methylprednisolone significantly decreased alveolitis and HA levels in the alveolar space and serum but did not affect fibrosis. In a separate study by Li et al., irradiation to the lower portion of the right lung of rats induced an accumulation of HA in BAL that was significantly elevated (152.8 *μ*g/L) 6 weeks after irradiation compared to untreated controls (5.5 *μ*g/L) [115]. Interestingly, HA BAL levels were not elevated at earlier (2 and 4 weeks) or later (8 and 10 weeks) time points. HAS2 gene expression was elevated at 4, 6, and 10 weeks of irradiation while Hyal2 expression decreased concomitantly. In a third study by Iwakawa et al. histological analysis of HA lung levels at sites of inflammation was evident within 12 hrs of radiation exposure and resolved 2 weeks later [116].

### 4.8. Ventilation

Early respiratory distress syndrome (RDS) in premature infants is characterized by lung edema ultimately leading to fibrosis or bronchopulmonary dysplasia [117]. Testing the hypothesis that increased HA levels in the alveolar interstitium would be associated with severe RDS; Juul et al. subjected 34 preterm delivered Macaca nemestrina monkeys to ventilation and found that HA levels were elevated (86.3 *μ*g/g lung wet weight) in lung extracts with progressively more severe RDS compared to animals without RDS (19.6 *μ*g/g) (*p* < 0.001) (radiometric assay) [117]. As the severity of RDS increased, HA was increasingly associated with the microvasculature in the interalveolar spaces, and in the most severe cases, HA was present in the alveolar walls. In a separate study by Bai et al., HAS3 −/− mice demonstrated reduced neutrophil infiltration, macrophage inflammatory protein-2 production, and lung microvascular leakage in response to ventilator-induced lung injury [118]. The HA produced by WT mice in response to ventilator-induced injury contained both HMW (1600 kDa) and LMW (<360 kDa) HA while only HMW HA was observed in HAS3 −/− mice. Wang et al. described a therapeutic improvement in ventilated premature piglets when surfactant treatment was supplemented with HA [119].

### 4.9. Bacterial Infection

Bacteria colonize the respiratory tract by multiplying in or on the airway epithelial mucosa, causing inflammation, increased mucus secretion, and impaired mucociliary clearance [120]. In one report by Juul et al., neonatal piglet lung HA levels decreased 4 hrs after inoculation with group B streptococcus (27 *μ*g/g wet weight) and atelectasis plus pneumonia (10 *μ*g/g) compared to control piglets (51 *μ*g/g) (*p* < 0.005) (radiometric assay) [121]. Later time points were not examined. In a study conducted by van der Windt et al., enhanced pulmonary inflammation was associated with decreased* Klebsiella pneumoniae* growth in CD44 −/− mice compared to WT mice [122]. Lethal dosage with this bacterium did not impact the survival of CD44 −/− mice compared to WT mice, though resolution of lung inflammation was delayed. Other studies confirmed and expanded some of these observations [123, 124]. Marion et al. provided evidence that* Streptococcus pneumoniae* have the capacity to utilize HA as a carbon source during colonization [125]. Intranasal exposure of staphylococcal enterotoxin B induced elevated levels of HA in BAL (~40 pg/mL) compared to control mice (~18 pg/mL) (ELISA-like assay) [126] and treatment with an inhibitor of HA synthesis (4-methylumbelliferone) had a protective effect on lung injury caused by this toxin [127]. Chang et al. demonstrated that intratracheal inoculation with* Escherichia coli* caused a rapid (2 hr) induction of HAS1 and HAS2 gene expression associated with increased histological staining for HA in the lungs [128].

### 4.10. Conclusions

These studies indicate the following. (i) The induction of HA synthesis in the lung is an early event following lung injury, occurring within hours of the original stimulus. (ii) HA synthesis precedes pulmonary fibrosis, and HA levels continue at elevated levels throughout the initial stages of fibrosis. (iii) The clearance of HA matrices following pulmonary injury is necessary for the proper resolution of inflammation. (iv) The size of HA is affected by the stage of disease and can exacerbate respiratory symptoms following injury (Figure 2). (v) The covalent modification of HA with heavy chains from inter-a-inhibitor, by the enzyme TSG-6, plays a key role in the development of airway inflammation [15–17, 72, 73] (Figure 2).

## 5. Overall Summary and Conclusions

The rise and fall of HA levels in the injured lung are essential for its repair and return to homeostasis (Figure 1). The data reviewed in this paper present a model whereby HA synthesis in the airways can be induced by either an acute injury (i.e., an asthma exacerbation) or a series of chronic insults (i.e., exposure to environmental irritants, smoking, lung transplant, genetic diseases, etc.). The induction of HA synthesis in lung tissue following an acute injury can be relatively rapid, occurring within the first 24 hrs of injury [74]. Once induced by injury, lung HA levels can remain elevated for several weeks [74]. Chronic conditions induce a low-level, long-term injury that leads to the accumulation of abnormally high levels of HA in the lung tissue. Following both acute and chronic pulmonary injury, two modifications happen to HA that regulate its pathobiology: (i) the covalent transfer of heavy chains from inter-alpha-inhibitor to the C6 hydroxyl of an N-acetylglucosamine residue on HA and (ii) its degradation into LMW fragments (Figure 2). The former is a process regulated by the enzyme TSG-6 [14, 15, 17] while the latter is regulated by the activity of hyaluronidases and the production of free radicals at the site of injury [6, 9].

Clearly there is a connection between elevated HA levels and its regional distribution with inflammatory cell infiltration. Leukocytes are typically found embedded within HA matrices of the airway submucosa and in perivascular regions. The covalent modification of HA with heavy chains from inter-alpha-inhibitor has been shown to promote leukocyte adhesion to HA matrices [77, 129], and this modification has been described in several respiratory disorders [15–17, 72, 73]. The effect that this modification has on leukocyte pathobiology remains to be established and both proinflammatory and anti-inflammatory data have been reported [1, 2, 15, 130].

While the induction of HA synthesis is clearly triggered by pulmonary injury, its role in directing fibrotic events remains to be defined and the signals that orchestrate its turnover and degradation following injury are not fully understood. The production of HA fragments, as a result of matrix remodeling and tissue damage by free radicals, is one of the signals that mediates inflammation and fibrosis [3, 45]. These HA “danger signals” operate via TLR4, MyD88, and TIRAP signaling pathways in the airways where they regulate AHR and the production of proinflammatory cytokines [111]. Intratracheal instillation of LMW HA fragments induces CD44-dependent AHR while instillation of HMW HA is protective [39]. Thus, in the airways, and other biological systems, the size of HA is one of the mechanisms whereby this relatively simple polysaccharide directs inflammatory and fibrotic events.

A variety of stimuli have been found to induce the accumulation of HA in respiratory secretions, reaching levels between 27 and 547 *μ*g/L in BAL fluid. This is in contrast to the relatively low levels of HA found in the respiratory secretions of healthy controls which ranged from 0 to 53 *μ*g/L in the BAL fluid reported in this review. The variation of HA levels in the BAL of healthy controls cannot be explained by difference in analytical techniques since no trend was observed between these techniques in that regard. It is more likely that the selection criteria of a particular healthy control patient cohort and/or the volume of BAL fluid instilled and collected may be responsible for the range of HA levels observed in healthy controls. The cellular source of HA found in respiratory secretions includes the airway epithelium [131], and the serous epithelial cells of the submucosal glands [132, 133], while it appears to be a minor component of goblet and mucous gland cell secretions [133]. The contribution that HA makes to respiratory secretions is not fully understood, though its large hydrodynamic volume is likely to contribute to mucus hydration and its viscoelastic properties.

While elevated levels of HA promote pulmonary wound healing in acute injury, it is less clear whether elevated levels of HA promote wound healing in a chronic state. In allergic asthma, where the respiratory system mounts an immune response against a relatively inert “invader,” it is not clear whether the induction of HA has beneficial or harmful effects. If HA is exerting a beneficial effect in a specific respiratory disease, then therapeutic strategies to enhance this effect might accelerate and improve the healing process. Indeed, several reports have described beneficial effects in the administration of HA, itself, as a therapy for several pulmonary conditions [19, 95, 97, 98, 107, 108, 119, 134–150], though the mechanisms whereby these beneficial effects occur remain to be defined. If HA is exerting a negative effect, such as might be the case in a chronic or allergic condition, then therapeutic strategies that antagonize HA synthesis, binding proteins, receptors, and so forth would be more effective.

In summary, these data present HA as a unique polysaccharide matrix which contributes to the homeostasis, maintenance, and repair of the injured lung (Figure 2). The biochemical and biophysical properties of HA endow this polysaccharide with protective and regenerative effects that contributes to edema and the regulation of AHR. The accumulation of HA at sites of pulmonary injury and repair provides an essential microenvironment that directs inflammatory events and fibrosis. The failure to mount an effective immune response, the inability to resolve inflammation, and/or the development of irreversible fibrosis in the respiratory system is, in part, influenced by the regulation of this relatively simple glycosaminoglycan.
